# Active DNA Demethylation in Plants

**DOI:** 10.3390/ijms20194683

**Published:** 2019-09-21

**Authors:** Jara Teresa Parrilla-Doblas, Teresa Roldán-Arjona, Rafael R. Ariza, Dolores Córdoba-Cañero

**Affiliations:** 1 Maimónides Biomedical Research Institute of Córdoba (IMIBIC), 14071 Córdoba, Spain; b52padoj@uco.es (J.T.P.-D.); ge2roarm@uco.es (T.R.-A.); ge1roarr@uco.es (R.R.A.); 2 Department of Genetics, University of Córdoba, 14071 Córdoba, Spain; 3 Reina Sofía University Hospital, 14071 Córdoba, Spain

**Keywords:** epigenetics, DNA methylation, 5-methylcytosine, base excision, DNA repair, DNA glycosylases, gene imprinting, transposons, biotic stress, abiotic stress

## Abstract

Methylation of cytosine (5-meC) is a critical epigenetic modification in many eukaryotes, and genomic DNA methylation landscapes are dynamically regulated by opposed methylation and demethylation processes. Plants are unique in possessing a mechanism for active DNA demethylation involving DNA glycosylases that excise 5-meC and initiate its replacement with unmodified C through a base excision repair (BER) pathway. Plant BER-mediated DNA demethylation is a complex process involving numerous proteins, as well as additional regulatory factors that avoid accumulation of potentially harmful intermediates and coordinate demethylation and methylation to maintain balanced yet flexible DNA methylation patterns. Active DNA demethylation counteracts excessive methylation at transposable elements (TEs), mainly in euchromatic regions, and one of its major functions is to avoid methylation spreading to nearby genes. It is also involved in transcriptional activation of TEs and TE-derived sequences in companion cells of male and female gametophytes, which reinforces transposon silencing in gametes and also contributes to gene imprinting in the endosperm. Plant 5-meC DNA glycosylases are additionally involved in many other physiological processes, including seed development and germination, fruit ripening, and plant responses to a variety of biotic and abiotic environmental stimuli.

## 1. Introduction

DNA methylation at carbon 5 of cytosine (5-methylcytosine, 5-meC) is a stable but reversible modification, usually associated with gene silencing, that functions as an epigenetic mark in embryonic development, X-chromosome inactivation, imprinting, and control of transposon activity [1]. DNA methylation is established and maintained by DNA methyltransferases, which transfer a methyl group from S-adenosyl-L-methionine to carbon 5 of cytosine to generate 5-meC [2]. Mammalian methylation primarily occurs at symmetric CG contexts in patterns that are established by the DNA methyltransferase 3 (DNMT3) family of de novo methyltransferases and copied in post-replicative hemimethylated DNA by the maintenance methyltransferase DNMT1 [2]. Plant DNA methylation can occur at any cytosine sequence context: CG, CHG, or CHH (H = A, T, C). In plants, de novo methylation is usually mediated by the RNA-directed DNA methylation (RdDM) pathway and catalyzed by the DNMT3-like enzymes DRM1 and DRM2 [3]. However, at some genomic regions DNA methylation is established in a RdDM-independent manner by two members of the plant-specific chromomethylase family (CMT2 and CMT3) [4,5]. Plant DNA methylation is maintained by different pathways depending on the sequence context. At CG sites it is maintained by the DNMT1 ortholog MET1, at CHG sites by CMT2 and CMT3 [3], and at CHH sequences by persistent RdDM-dependent de novo methylation catalyzed by DRM2 [3] or RdDM-independent methylation carried out by CMT2 [4]. 

DNA methylation patterns, which change during normal development, are the dynamic outcome of antagonistic methylation and demethylation processes. DNA demethylation may be either the passive result of consecutive DNA replication cycles in the absence of maintenance methylation or an active mechanism involving enzymes that remove 5-meC [6]. Despite intense research efforts, the nature of DNA demethylation mechanisms in animals has been largely elusive [7]. However, accumulating evidence suggests that a family of dioxygenases (Ten-Eleven Translocation, TET proteins) convert 5-meC into derivatives that inhibit maintenance methylation, thus promoting passive demethylation, and/or are actively removed through DNA repair [8]. 

In contrast to animals, plants have evolved a specific active DNA demethylation mechanism involving 5-meC DNA glycosylases that directly excise 5-meC and initiate its replacement with unmethylated C through a base excision repair (BER) pathway [9,10]. Plant 5-meC DNA glycosylases are typified by *Arabidopsis* ROS1 (REPRESSOR OF SILENCING 1) [11,12,13] and its paralogs DME (DEMETER) [12,14], DML2, and DML3 (DEMETER-LIKE proteins 2 and 3) [12,15,16]. ROS1, DML2, and DML3 counteract excessive methylation at several hundred loci across the genome in vegetative tissues [15,16,17]. DME demethylates the maternal allele of imprinted genes in the endosperm [18], but its basal function appears to be the reactivation of transposons in companion cells of male and female gametophytes to generate siRNAs that would reinforce transposon silencing in male and female gametes [19,20]. In addition to gametogenesis and seed development, active DNA demethylation serves important functions during various stages of the plant life cycle, including seed germination, fruit ripening, and responses to a variety of stress conditions [21,22,23,24,25].

BER-mediated DNA demethylation in plants is a complex process comprising three major steps: (i) excision of 5-meC coupled to strand incision, which generates a gapped DNA intermediate with a 3′-blocked end; (ii) cleaning-up of the 3’-terminus to generate a 3´-OH group; (iii) gap DNA repair synthesis inserting an unmethylated C and sealing of the DNA strand by DNA ligation. In addition to 5-meC DNA glycosylases, the plant DNA demethylation pathway involves a variety of enzymes processing the successive DNA demethylation intermediates, as well as other proteins performing regulatory roles. 

In this review we summarize the key molecular features of the enzymes responsible for 5-meC removal, describe the roles of additional proteins implicated in the various stages of the plant DNA demethylation pathway and its regulation, and outline the main physiological processes in which active DNA demethylation has been implicated.

## 2. The DML Family: Plant-Specific DNA Glycosylases That Excise 5-meC

Plant 5-meC DNA glycosylases are grouped in the DEMETER-LIKE (DML) family, which belongs to the HhH-GPD superfamily, the largest and most functionally diverse group of DNA glycosylases [26]. DML proteins are unusually large DNA glycosylases, ranging from 1100 to over 2000 residues and so far they have been only detected in plants, including mosses (e.g., *Phycomitrella patens*) and unicellular green algae (e.g., *Ostreococcus*), suggesting that active demethylation through 5-meC excision arose early during plant evolution [9]. DML proteins are bifunctional enzymes with both DNA glycosylase and apurinic/apyrimidinic (AP) lyase activities [12,13,15,16,18].

**Figure 1:**
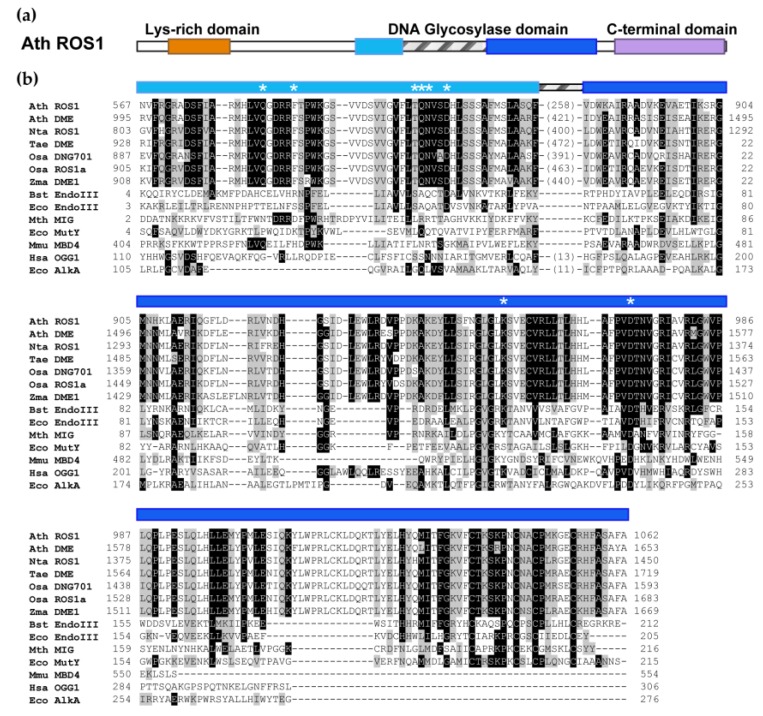
Bipartite DNA glycosylase domain in members of the DEMETER-LIKE (DML) family. (**a**) Schematic representation of ROS1 domains conserved among DML proteins. (**b**) Multiple sequence alignment of DML and HhH-GPD representative proteins. Critical residues for catalysis or DNA binding in ROS1 (REPRESSOR OF SILENCING 1) are indicated by asterisks (see text for more details). Names of organisms are abbreviated as follows: *Ath, Arabidopsis thaliana; Nta, Nicotiana tabacum; Ta, Triticum aestivum; Osa, Oryza sativa; Zma, Zea mays; Bst, Bacillus stearothermophilus; Eco, Escherichia coli; Mth, Methanobacterium thermoautotrophicum; Mmu, Mus musculus; Hsa, Homo sapiens.* Genbank accession numbers are: *Ath* ROS1: AAP37178; *Ath* DME: ABC61677; *Nta* ROS1: BAF52855; *Tae* DME: AEF38424; *Osa* DNG701: B8YIE8; *Osa* ROS1a: XP_015650531; *Zma* DME1: AQK70999; *Bst* EndoIII: 1P59; *Eco* EndoIII: P20625; *Mth* Mig: NP_039762; *Eco* MutY: NP_417436; *Mmu* MBD4: 1NGN; *Hsa* OGG1: O15527; *Eco* AlkA: P04395.

Sequence alignment with other members of the HhH-GPD superfamily revealed that DML proteins possess a discontinuous DNA glycosylase domain comprising two segments connected by a predicted disordered region of variable sequence and length [27] (Figure 1). The function of this region is unknown, although it has been speculated that it might facilitate target localization and undergo folding upon DNA binding [27]. Replacement of this linker region in DME with a short peptide sequence does not abrogate 5-meC excision, indicating that is dispensable for enzymatic activity [28]. 

DML proteins show in their catalytic domain a characteristic helix–hairpin–helix motif and a Gly/Pro rich loop (HhH-GPD, amino acids from 941 to 969 in ROS1), followed by a conserved catalytic aspartate (amino acid 971 in ROS1) [26]. In addition, they share a lysine residue specifically conserved in bifunctional DNA glycosylases (amino acid 953 in ROS1) and a [4Fe-4S] cluster with four cysteine residues (Cys-X_6_-Cys-X_2_-Cys-X_5_-Cys) conserved in a subset of HhH-GPD proteins (amino acids from 1309 to 1055 in ROS1) [29] (Figure 1). It has been demonstrated that the invariant aspartate of the HhH-GPD motif is essential for catalytic activity [12,16,18]. Using *Bacillus stearothermophilus* Endo III as a template for a 3D model structure, additional residues specifically involved in 5-meC recognition and catalysis have been identified in ROS1 [27]. Residues T606 and D611, located in the first segment of ROS1 catalytic domain and predicted to be positioned between the base stack and the recognition pocket, were found to be essential for DNA glycosylase activity. On the other hand, mutational changes in two aromatic residues presumably located in the substrate-binding pocket (F589 and Y1028) altered the base specificity of the enzyme. Additionally, amino acid Q607, which is essential for both catalytic activity and stable DNA binding, has been proposed as a base flipper, replacing 5-meC in the base stack when it is extruded from the helix and inserted in the substrate-binding pocket [27]. Interestingly, Q607 is necessary for efficient extrusion of both methylated and unmethylated bases, which suggests that the enzyme uses helix-invading residues to actively interrogate DNA in search of 5-meC [30]. Furthermore, Q607 and two residues positioned close to the G opposite 5-meC (R903 and M905) are specifically required for efficient excision of 5-meC opposite G, but not for mismatched 5-meC [30]. On other hand, point mutations at each cysteine residue of the [4Fe-4S] cluster motif of DME resulted in abrogation of DNA glycosylase activity, suggesting that this motif is also necessary for catalytic activity and/or protein stability [28]. It has been proposed that the oxidation state of the [4Fe-4S] cluster mediates binding and dissociation of DML proteins from DNA [31].

In addition to their bipartite DNA glycosylase domain, DML proteins are characterized by a large C-terminal domain exclusively conserved among DML family members and a short N-terminal domain significantly rich in lysine [27,32]. The N-terminal basic domain is not essential for catalytic activity [28,32], but in ROS1 mediates methylation-independent binding to DNA and endows the protein with the capacity to slide along DNA substrates in search of 5-meC [32,33]. The C-terminal domain is highly conserved among DML family members, but no sequence similarity has been detected with any known protein in either plants or other taxa. Point mutations or deletions in the C-terminal domain of DME and ROS1 result in abrogation of the 5-meC excision activity [13,28,32]. The C-terminal domain of ROS1 lacks detectable enzymatic activity and binds DNA with very low affinity [32], whereas its isolated DNA glycosylase domain is inactive for 5-meC excision but retains partial AP lyase activity [34]. Interestingly, when the C-terminal domain is added in trans to the DNA glycosylase domain, the base excision activity is restored, suggesting that the C-terminal region is essential for the 5-meC DNA glycosylase activity, perhaps by stabilizing the DNA glycosylase domain and/or stimulating its enzymatic activity [34]. 

## 3. The Plant DNA Demethylation Pathway 

### 3.1. Base Excision and Strand Incision

**Figure 2:**
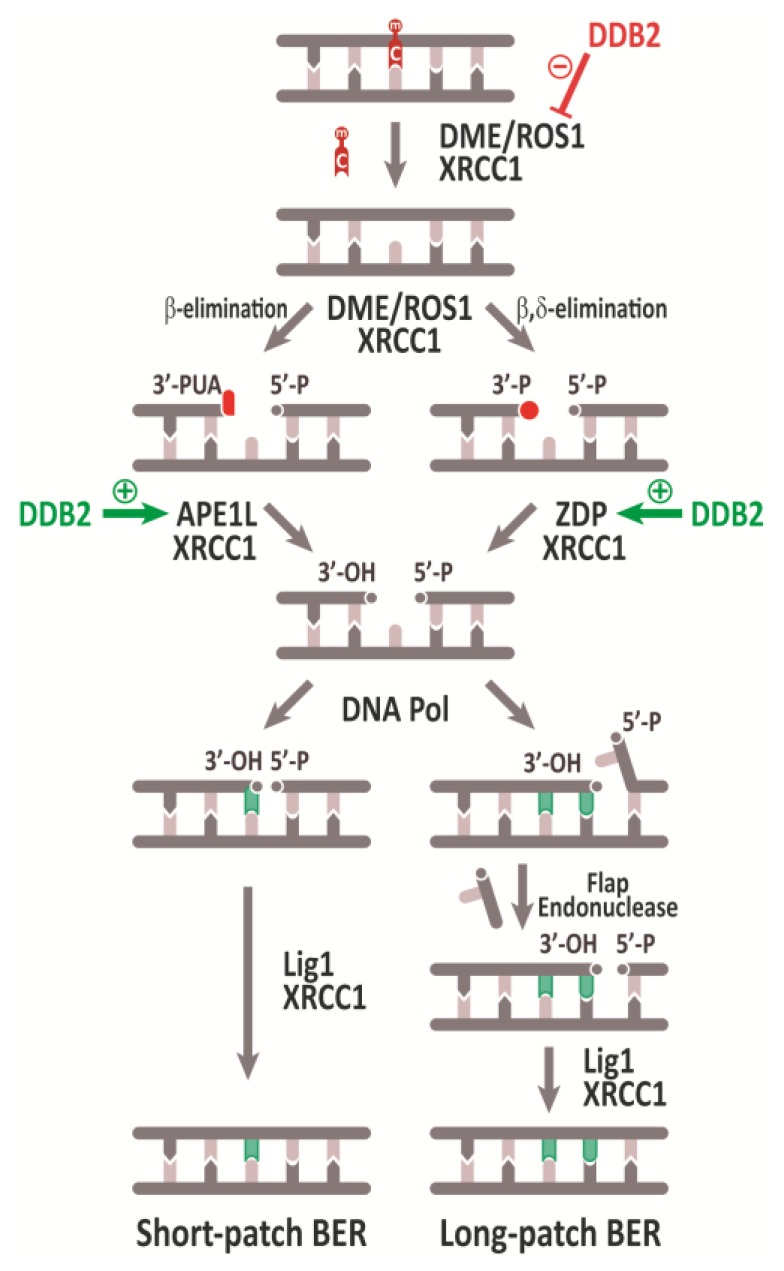
Schematic representation of the active DNA demethylation pathway in *Arabidopsis*. Different 3’ blocked ends are indicated in red. Stimulatory or inhibitory effect of DDB2 over different proteins of active DNA demethylation pathway are indicated with green or red arrows, respectively. See text for details.

Plant 5-meC DNA glycosylases initiate BER-mediated DNA demethylation by hydrolyzing the glycosylic bond between 5-meC and the deoxyribose moiety. Upon catalyzing 5-meC excision, they cleave the phosphodiester backbone 3′ to the generated AP site by β- or β, δ-elimination. The result is a single-nucleotide gap flanked by 3′-PUA or 3′-P ends, respectively, and a 5′-P terminus (Figure 2) [12,13,15,16,18]. It has been reported that in ROS1 5-meC excision is coupled to the AP lyase step [35], in agreement with a unified mechanism of DNA glycosylases/AP lyases postulating coordinated base excision and strand incision as a result of Schiff base formation [36]. In fact, excision of 5-meC catalyzed by ROS1 and DME proceeds via a Schiff base intermediate [12]. 

ROS1, DME, and DML3 target 5-meC in any sequence context, although they display a preference for CG sequences [12,15,16]. Mutational analysis of ROS1 supports a mechanism in which the enzyme detects 5-meC by extrahelical interrogation [30] (see Section 2 above). Importantly, ROS1 excises 5-meC less efficiently when correctly paired with G than when mispaired with A, C, or T [35], which suggests that extrusion of the target base from the DNA helix is a limiting step in 5-meC excision. After performing 5-meC excision and strand incision in a CG context, ROS1 tightly binds its reaction product, thus inhibiting processing of the 5-meC in the opposite strand [32,35]. It has also been shown that the presence of an abasic site instead of 5-meC at a CG site strongly inhibits DME activity on the 5-meC located in the complementary strand [18]. It is likely that strong product binding and the requirement for two intact DNA strands for 5-meC excision contribute to avoiding deleterious double strand breaks in symmetric DNA methylation contexts (CG and CHG).

Interestingly, in addition to 5-meC, DML proteins also efficiently excise T (=5-meU) but only when mispaired with G [12,16,35]. This strongly suggests that DML proteins additionally perform a DNA repair function, preventing the mutagenic consequences of spontaneous deamination of 5-mC:G pairs to T:G mispairs. 

### 3.2. Gap Tailoring by 3´-End Cleaning

The product generated after 5-meC excision and strand incision catalyzed by DML proteins is a single nucleotide gap with either 3´-PUA or 3´-P ends (Figure 2). These non-canonical 3´ termini must be converted to 3´-OH ends before DNA polymerase and ligase activities complete the DNA demethylation process.

In *Arabidopsis*, strong biochemical and genetic evidence indicates that 3′-P processing is mainly performed by the DNA phosphatase ZDP, both in active DNA demethylation [37] and during BER of different DNA lesions [38,39]. ZDP accounts for all DNA phosphatase activity detected in *Arabidopsis* cells in vitro and cell-free extracts from *zdp* mutant plants are unable to complete DNA demethylation [37]. Mutations in *ZDP* cause hypermethylation at hundreds of genome loci and, importantly, ZDP and ROS1 interact in vitro and colocalize in vivo [37]. On other hand, the *Arabidopsis* AP endonuclease APE2 possesses limited 3´-phosphatase activity and may function redundantly with ZDP during active DNA demethylation [40]. 

Blocked 3′-PUA termini generated by β-elimination need to be processed by a 3′-phosphodiesterase activity. Like human APE1 [41], two *Arabidopsis* AP endonucleases, APE1L and ARP, possess 3´-phosphodiesterase activity and are able to convert 3´-PUA into 3´-OH ends [42,43]. However, APE1L displays a much more potent activity than ARP against 3´-PUA termini and in vitro and in vivo interactions have only been reported between APE1L and ROS1 [42]. Although both *ape1l* and *arp* mutant plants display an altered methylome, the number of differentially methylated regions in *ape1L* mutants is about 10-fold higher than in *arp* mutants [42]. Therefore, it has been suggested that the majority of 3´-PUA intermediates generated during active DNA demethylation are processed by APE1L [42]. Importantly, whereas single *zdp* and *ape1l* mutants exhibit a normal phenotype, double *zdp ape1l* mutants are maternally lethal, indicating that ZDP and APE1L function downstream of 5-meC DNA glycosylases/lyases in independent branches of the active DNA demethylation pathway [42] (Figure 2). 

### 3.3. Gap Filling and DNA Ligation

Once noncanonical 3´ termini have been converted to 3´-OH ends, DNA polymerase and DNA ligase activities must complete the DNA demethylation process. In mammals, gap filling during BER occurs either by insertion of one nucleotide (short-patch, SP-BER) or by incorporation of two or more nucleotides and simultaneous strand displacement (long-patch, LP-BER) [44]. Although both SP- and LP-BER subpathways have been detected in repair reactions catalyzed by *Arabidopsis* cell-free extracts [45], the DNA polymerase(s) responsible for repair synthesis remain(s) unknown. In mammals, single-nucleotide insertion during SP-BER is catalyzed by the X-family DNA polymerase β (Pol β) [46], which also incorporates the first nucleotide during LP-BER, while subsequent elongation is carried out by replicative DNA polymerases (DNA Pol δ and Pol ε) [47]. No homologs of DNA Pol β have been detected in plants, where the only X-family member is DNA polymerase λ (Pol λ). Although Pol λ has been partially characterized in rice and *Arabidopsis* and reported to be implicated in oxidative lesion bypass and repair of UV-induced DNA damage [48,49,50], its role in BER, and specifically in active DNA demethylation, remains to be determined. 

Strand displacement during LP-BER generates a 5´-flap structure that in mammalian cells is removed by FEN1, a structure-specific DNA endonuclease that also performs an essential role processing Okazaki fragments during DNA replication [51]. Mutation of the *Arabidopsis* FEN1 homolog causes hypersensitivity to DNA-damaging agents and telomere shortening [52], but its role in plant BER and/or DNA demethylation has not yet been established.

Both the SP- and LP-BER DNA demethylation subpathways generate a single DNA strand break (SSB) that needs to be sealed by a DNA ligase activity (Figure 2). In mammals, a complex of Lig III and the scaffold protein XRCC1 catalyzes DNA ligation in SP-BER [53], whereas LP-BER ligation is performed by Lig I [54]. The *Arabidopsis* genome encodes three DNA ligases (LIG1, LIG4, and the plant-specific LIG6) but lacks a Lig III homologue [55]. Plant DNA ligases have been implicated in different processes [56,57,58], but only *Arabidopsis* LIG1 has been demonstrated to participate in BER, performing the ligation step in both SP- and LP-BER subpathways [59]. *Arabidopsis* LIG1 colocalizes with ROS1, ZDP, and APE1L in vivo and is essential for DNA demethylation and activation of maternally imprinted genes *FWA* and *MEDEA* in the endosperm [60]. Moreover, gene interaction analyses indicate that LIG1 participates in DME-mediated demethylation [61]. Although *Arabidopsis* XRCC1 lacks the BRCT2 domain that in mammals is implicated in the interaction with DNA ligase III, for which there is no homolog in plants, it stimulates the ligation step during ROS1-initiated DNA demethylation, probably through interaction with LIG1 [62]. In addition, XRCC1 interacts in vitro with both ROS1 and ZDP and stimulates their respective activities [62]. Therefore, XRCC1 functions at different stages during active DNA demethylation in plants (Figure 2).

### 3.4. Regulation of Active DNA Demethylation

It is still poorly understood how plant 5-meC DNA glycosylases are directed to specific genomic regions, but the process likely involves the presence of particular chromatin modifications at target loci and/or the activity of recruiting factors. It has been reported that loci targeted by ROS1 are enriched for H3K18Ac and H3K27me3 and depleted of H3K27me and H3K9me2 [63]. On other hand, a genetic screen for DNA hypermethylation mutants identified IDM1 (INCREASED DNA METHYLATION 1) as a histone acetyltransferase acting on H3K18 and required for demethylation at a subset of ROS1-targeted loci [64]. Furthermore, it has been shown that IDM1 forms a complex with additional proteins, including IDM2, IDM3, the METHYL-CPG-BINDING DOMAIN-CONTAINING PROTEIN 7 (MBD7), and the HARBINGER TRANSPOSON-DERIVED PROTEINS HDP1 and HDP2 [65,66,67]. It has been proposed that such an IDM complex facilitates ROS1 recruitment by IDM1-catalyzed histone acetylation [67], but the specific mechanism involved remains unknown. On other hand, the RNA-binding protein ROS3 is required for ROS1-dependent demethylation at several genomic regions [68], suggesting that small RNAs similar to those targeting RdDM may be involved in recruiting ROS1 to specific loci. However, the role of ROS3 in targeting DNA demethylation, if any, remains undetermined. 

Plant DNA methylation and demethylation mechanisms need to be balanced in order to maintain stable genome-wide methylation patterns. *Arabidopsis* ROS1, DML2, and DML3 antagonize RdDM targeted to transposable elements (TE) and TE-derived sequences and prevent methylation spreading to nearby genes [15,16,17,69] (see Section 4.1 below). Interestingly, *ROS1* expression is repressed in mutants defective in RdDM or MET1 [70,71,72,73,74], which suggests that DNA methylation and demethylation are coordinated processes. In fact, methylation of a short sequence targeted by RdDM in the *ROS1* promoter is required for *ROS1* expression [75,76]. This sequence, which has been defined as a “methylstat” that senses DNA methylation levels, is conserved in other plant species [76], suggesting that it has evolved as a mechanism to balance methylation and demethylation processes. An additional factor implicated in such DNA demethylation control is DDB2 (DNA damage-binding protein 2). DDB2 was initially identified in human cells as a DNA repair factor in complex with DDB1 and is involved in recognition of DNA lesions, probably acting as a sensor for conformational changes in DNA [77]. *Arabidopsis ddb2* mutant plants show genome-wide methylation alterations [78] and it has been shown that DDB2 forms a complex in vivo with ROS1 and AGO4, controlling RdDM at the ROS1 locus and influencing its expression [79]. Moreover, biochemical evidence suggests that DDB2 plays an additional role in regulating various steps of ROS1-initiated DNA demethylation. Thus, it interacts in vitro with ROS1, inhibiting its 5-meC excision activity, but also with ZDP and APE1L, stimulating their enzymatic (3´-end cleaning) activities, thus avoiding accumulation of toxic SSB intermediates with blocked 3′-ends [79]. Altogether, these findings suggest that the coordinated control of DNA demethylation involves complex mechanisms at both the transcriptional and posttranscriptional levels. 

There are also indications that active DNA demethylation may be further integrated with important aspects of the cell metabolism through the conserved [4Fe-4S] cluster conserved in DML proteins (see Section 2 above). Thus, genetic screens aimed at finding new factors involved in DME- or ROS1-initiated DNA demethylation have identified at least four proteins (AE7, NAR1, DRE2, and MET18/MMS19) functioning in the cytosolic iron–sulfur assembly (CIA) pathway, which is responsible for the maturation of cytosolic and nuclear Fe-S proteins [80,81,82,83,84]. Therefore, controlled assembly of [4Fe-4S] clusters in 5-meC DNA glycosylases may represent an important link connecting plant metabolism and nutrition states with DNA methylation patterns. 

## 4. Biological Roles of DNA Demethylation

Plant DNA methylation is targeted to TEs and other repetitive sequences. Long TEs are primarily located in heterochromatin and methylated by CMT2 and CMT3, but gene-rich euchromatic regions contain a significant portion of shorter TEs or TE-derived sequences that are targeted by RdDM [4,85,86]. A primary function of plant DNA demethylation is to maintain genome stability by counteracting RdDM, thus preventing methylation spreading to neighboring genes. Active DNA demethylation is also implicated in genome-wide methylation changes during reproductive development that occur in both the female and male gametophytes. Demethylation in gametophytes, which takes places in companion cells and is also mostly directed to euchromatic TE sequences, may be important for reinforcing methylation patterns in gametes and one of its consequences is gene imprinting in the endosperm [19,20]. Additional demethylation associated to reproductive development takes place during seed development and germination, as well as during fruit ripening. A third major role of DNA demethylation in plants is to activate genes in response to biotic or abiotic stimuli, in many cases by targeting TE sequences located at their 5´ regions. 

### 4.1. Maintenance of Genome Stability by Preventing Hypermethylation

*Arabidopsis* ROS1, DML2, and DML3 are expressed in a wide range of plant vegetative tissues and they seem to contribute to the stability and plasticity of plant epigenome and protect the genome from excessive methylation [11,15,16,17,63]. *Arabidopsis* ROS1, which is the major 5-meC DNA glycosylase in vegetative tissues, was identified in a screen for mutants with deregulated expression of the repetitive RD29A-LUC transgene [11]. Whereas in wild-type plants the transgene and the homologous endogenous gene are expressed, *ros1* mutant plants display transcriptional silencing and hypermethylation of both loci [11], showing that active DNA demethylation can protect genes from being incorrectly silenced. Nearly 5000 regions are hypermethylated in the *ros1* mutant when compared to the wild-type, mainly located at TEs and intergenic regions [63,64]. Transposons targeted by ROS1 are usually close to protein-coding genes, and hypermethylation in *ros1* mutants spreads from TE edges to neighboring sequences [17,63]. These findings indicate that DNA demethylation by ROS1 aids in delimiting transposons and genes, preventing DNA methylation spreads from TEs, and, therefore, protecting nearby genes from transcriptional repression [63]. Analysis of hypermethylated regions in *ros1* mutants led to the identification of thousands of RdDM targets, and *ROS1* expression was reduced in all known RdDM mutants, suggesting that ROS1 counteracts DNA methylation established by the RdDM pathway, although it has been reported that it also antagonizes RdDM-independent DNA methylation at some loci [63]. The role of ROS1 in counteracting RdDM seems to be evolutionary conserved, since rice mutants deficient in the ROS1 homolog DNG701 also show hypermethylation at TEs [87]. ROS1 activity may be also important for reactivating expression of TEs that perform regulatory functions during vegetative development. For example, *ros1* mutant plants show a defect in the pattern of leaf epidermal cells characterized by a higher number of stomate lineage cells due to hypermethylation of a TE located at the promoter of *EPF2* (*EPIDERMAL PATTERNING FACTOR 2*) [88].

ROS1 might also contribute to genome stability by controlling telomere length. A study that characterized a mutation in the gene encoding the largest subunit of replication factor C (RFC1) as a *ros1* suppressor reported that telomere length was longer in *ros1* mutants compared to wild-type, whereas telomeres were shorter in the *rfc1* mutant [89]. These results suggest that ROS1 may play a role as a negative regulator of *Arabidopsis* telomere length, probably via regulation of TERT (telomerase reverse transcriptase) or other telomere-related proteins (POT1a, BT2), since expression levels of these proteins were slightly higher in *ros1* mutants and much lower in *rfc1* or *rfc1 ros1* mutants than in wild-type plants [89]. 

Similarly to ROS1, *Arabidopsis* DML2 and DML3 are expressed in a variety of plant organs and tissues and were found to be required for removing DNA methylation marks from improperly methylated cytosines [15,16]. Although *dml2* and/or *dml3* mutants do not show any obvious phenotypes, they display hypermethylation of cytosine residues that are either unmethylated or weakly methylated in wild-type plants. However, sites that are strongly methylated in wild-type plants are hypomethylated in *dml2* and/or *dml3* mutants [16]. Therefore, in addition to counteracting hypermethylation at erroneously methylated sites, DML2 and DML3 are required to maintain high methylation levels at properly methylated sites.

### 4.2. Methylome Reprogramming During Reproductive Development

The plant embryo is generated by fertilization of an egg cell by a sperm cell. In angiosperms, an additional fertilization event involving a second sperm cell and the central cell, a companion cell of the egg in the female gametophyte, generates an extraembryonic tissue, termed endosperm, that nourishes the embryo. The male gametophyte also has a companion cell, the vegetative cell, that forms a tube that transports the two sperm cells to the female gametophyte [90]. In *Arabidopsis*, active DNA demethylation initiated by DME in both types of companion cells, the vegetative, and central cells is important for successful reproduction and seed development.

*Arabidopsis* DME was identified in a search for mutations causing parent-of-origin effects on seed viability [14]. It was found to be necessary to establish imprinting of specific genes in the endosperm and its loss of function causes endosperm damage, alterations in embryonic development. and, therefore, abortive seeds [14]. DME-dependent DNA demethylation is initiated in the central cell of the female gametophyte [91], where the expression of the maternal alleles of *MEA*, *FWA*, and *FIS2* genes, among others, are activated prior to fertilization [14,18,92,93,94,95]. The presence of a wild-type maternal *DME* allele is essential for seed development and viability, since a maternally inherited *dme* mutation did not express *MEA::GFP* or *FWA::GFP* transgenes in the endosperm after fertilization, causing embryo and endosperm abortion even if the paternal *DME* allele is not mutated [14]. Analysis of the methylation levels of *Arabidopsis* wild-type endosperm showed hypomethylation of the maternal *MEA* or *FWA* alleles, in comparison with the paternal alleles, whereas in *dme* endosperm, maternal and paternal *MEA* alleles are highly methylated [18,92]. Altogether, these observations indicate that, in the central cell of *Arabidopsis*, DME DNA glycosylase is responsible for 5-meC removal to activate expression of maternally imprinted genes in the endosperm that are necessary for proper seed development.

Genome-wide DNA methylation profiles have revealed that DME-dependent demethylation in the endosperm is large-scale and causes extensive hypomethylation of TEs [94,96], thus suggesting that its basal function may not be gene imprinting. In fact, DME is also expressed in the companion cell of the male gametophyte, the vegetative cell, after separation of the sperm cells lineage and is required for pollen germination in some ecotypes [97,98]. In the vegetative cell, DME demethylates DNA to activate the expression of *MEA* and *FWA* genes and the *Mu1a* repetitive element, thus suggesting a function similar to that observed in the central cell [97]. A genome-wide comparison of hypomethylated regions in the vegetative cell and the maternal endosperm genome revealed a large overlap between sites demethylated in the male and female companion cells [20,94,96]. DME-dependent demethylation in companion cells affects preferentially small, AT-rich, euchromatic TEs [20]. Available evidence suggests that the basal function of DME appears to be the reactivation of TEs in companion cells to generate short interfering RNAs (siRNAs) that would reinforce transposon methylation in male and female gametes, thus promoting stable silencing of TEs in the embryo genome [19,20,99,100]. ROS1, DML2, and DML3 are expressed in the vegetative cell and may also contribute to such functions in male gametes [19].

Dynamic methylation reprogramming also takes place during subsequent seed development and germination. Comparison of time-series methylomes from dry and germinating *Arabidopsis* seeds revealed extensive gain of methylation in TEs at CHH sequences during seed development, followed by DML-independent, passive global CHH demethylation during germination [21]. During the late embryogenesis stage, ROS1 is implicated in active DNA demethylation at CG sequences, antagonizing RdDM in the embryo, and establishing specific DNA methylation in the endosperm [21].

Although mainly inferred through studies with *Arabidopsis*, the function of active DNA demethylation during plant reproductive development seems to be evolutionarily conserved. In rice (*Oryza sativa*), active DNA demethylation in the female gametophyte is also initiated in the central cell [91]. Rice lacks DME orthologues [101] but one of its four ROS1 orthologues, named ROS1a, seems to have similar functions to *Arabidopsis* DME in both male and female gametophytes, as observed in genetic and genome-wide DNA methylation studies. Thus, mutation in rice *ROS1a* causes loss of hypomethylation of the vegetative cell compared to the sperm, endosperm defects, and seed abortion [102,103]. In addition to ROS1a, rice DNG701, which is closely related to *Arabidopsis* DML2 and ROS1, seems to play a role in seed development, as *dng701* mutants show a high proportion of wrinkled seeds [87]. On other hand, maize (*Zea mays*) contains a *DME* homologous gene, termed *DME*-like, which is expressed at higher levels in the endosperm than in the embryo. Such differential expression correlates with lower DNA methylation levels in the endosperm at numerous genes implicated in seed development, including imprinted genes [104]. In wheat (*Triticum aestivum*) endosperm, the DME homolog TaDME demethylates the promoters of seed storage proteins, including immunogenic prolamins such as gliadins and low-molecular-weight glutenins (LMWgs), inducing their expression [105]. *TaDME* transcript suppression by RNAi entailed reduced accumulation of gliadins and LMWgs in the endosperm, suggesting a potential approach to generate celiac-suitable wheat cultivars [105].

Another stage of reproductive development in which active DNA demethylation performs important functions is fruit ripening. A global loss of DNA methylation takes place during tomato (*Solanum lycopersicum*) fruit ripening and has been recently attributed to active DNA demethylation mediated by *SlDML2*, one of the four putative *DML* genes encoding 5-meC DNA glycosylases in tomato (*SlDML1*, *SlDML2*, *SlDML3*, *SlDML4*) [22,23]. SlDML2 is an *Arabidopsis* ROS1 orthologue that is highly expressed during fruit ripening and that preferentially targets TEs in euchromatic regions. Tomato fruits in which *SlDML2* is silenced by RNAi [23] or mutated by using the CRISPR/Cas system [22] showed dramatic ripening inhibition due to an increase of DNA methylation of thousands of genes involved in fruit ripening. Interestingly, these genes included both ripening-induced genes (e.g., *RIN*, *NOR*, *PSY1*), which were repressed in *sldml2* mutants, and ripening-repressed genes (e.g., *CAP10B*, *RBCS-2A*), which were activated in *sldml2* mutants [22]. These observations suggest that SlDML2 DNA demethylase activity is necessary for both the activation of ripening-induced genes and the inhibition of ripening-repressed genes in order to promote tomato fruit ripening.

DNA methylation changes have been described in the ripening of other fruits by comparing the methylomes of immature and ripe fruits. Strawberry (*Fragaria vesca*) contains four *Arabidopsis ROS1* homologs (*FvDME1*, *FvROS1.1*, *FvROS1.2*, *FvROS1.3*) and, like tomato, showed decreased DNA demethylation during fruit ripening [106]. However, DNA hypomethylation during strawberry ripening was associated with downregulation of the RdDM pathway, as no upregulation of the DNA demethylases expression was observed [106]. By contrast, orange (*Citrus sinensis*), which also harbors four *Arabidopsis ROS1* orthologs (*CsDME*, *CsDML1*, *CsDML4*, *CsDML3*), showed a gradual global increase in DNA methylation levels during ripening correlated with a gradual decrease in DNA demethylases expression, since DNA methyltransferases transcripts were relatively less abundant and were not upregulated [107]. Interestingly, this DNA hypermethylation was associated with both repression and activation of several hundred genes.

### 4.3. Gene Regulation in Response to Biotic Stimuli

In the past few years, several studies have reported alterations in plant DNA methylation levels and the role of plant 5-meC DNA glycosylases in response to several biotic stimuli, such as association with symbiotic microorganisms and attack by diverse pathogens. For example, a recent report found that the symbiotic relationship of legumes with nitrogen-fixing soil bacteria is partially controlled by active DNA demethylation [108]. The legume *Medicago truncatula* possesses four putative members of the DML family (MtDME, MtDML1, MtROS, and MtROSL1). Only *MtMDE* is robustly upregulated in the differentiation zone of mature Rhizobium-induced nodules compared with roots [108]. *MtDME* expression is associated with upregulation of TEs close to nodule-specific cysteine-rich (*NCR*) genes. Nodules in wild-type plants displayed DNA hypomethylation in CG and CHG contexts compared to roots, mostly associated to *NCR* genes. Plants in which *MtDME* is silenced by RNAi exhibited smaller nodules incapable to fix nitrogen and showed hypermethylation and downregulation of 400 genes, including *NCR* genes, suggesting that MtDME is critical for nodule development [108].

There is also evidence that active DNA demethylation plays a role in antibacterial defense. Thus, *Arabidopsis ros1* mutants (but not *dml2* or *dml3* mutants) exhibited increased bacterial multiplication and propagation when infected with the pathogen *Pseudomonas syringae* [25]. Furthermore, they showed hypermethylation of an *AtREP4* helitron-related repeat located at the promoter of the *RGM1* disease resistance gene, concomitant with *RGM1* downregulation [25]. Several studies have also implicated DNA demethylation in responses to viral infections. For example, transcription levels of two *ROS1* homologs in *Nicotiana benthamiana* (*NbROS1* and *NbROS2*) decreased after local and/or global infection of the plant with different geminiviruses [109]. 

Several studies have reported that active DNA demethylation is also essential for defense against infection by pathogenic fungi. *Arabidopsis* triple *ros1 dml2 dml3* (*rdd*) mutant plants show enhanced susceptibility to the hemibiotrophic fungal pathogen *Fusarium oxysporum* and exhibit downregulation of many plant response stress genes containing TE sequences in their promoters, a subset of which display hypermethylation [110]. A subsequent study reported that ROS1 is the main enzyme responsible for demethylation of promoter-associated TEs and for the induction of defense-related genes in response to *F. oxysporum* infection [111]. Interestingly, the consequences of active DNA demethylation upon infection seem to depend on the fungal lifestyle. Thus, *Arabidopsis ros1* mutants exhibit increased susceptibility to the biotrophic fungus *Hyaloperonospora arabidopsidis*, but increased resistance to the necrotrophic fungi *Plectosphaerella cucumerina* and *Alternaria brassicicola* [112]. These contrasting outcomes are partially linked to differential effects of *ros1* deficiency on salicylic acid- and jasmonic acid-dependent signaling pathways, implicated in plant resistance to biotrophs and necrotrophs, respectively [112].

### 4.4. Gene Regulation in Response to Abiotic Stress

Many studies have reported changes in DNA methylation levels in response to a variety of abiotic stresses that correlate with transcriptional regulation of genes implicated in plant stress responses [24]. However, the role of the enzymes involved in active DNA demethylation on these alterations has been incompletely addressed and remains poorly understood. Reports in this field have been mostly limited to analyzing changes in DNA demethylases gene expression during different abiotic stress conditions and few studies have performed more comprehensive analyses involving, for example, loss-of-function mutants. 

One area that has received particular attention is the role of methylation/demethylation processes in nutritional stress. In *Arabidopsis*, zinc (Zn) starvation causes moderate changes in total methylation, leading to hypo- and hyper-methylation predominantly in TEs, promoters, and genes in CG and CHG contexts, preferentially in the proximity of transcriptionally responsive genes [113]. Mutant *ros1* plants are impaired in Zn uptake under Zn deficiency, suggesting that DNA demethylation is important for Zn starvation tolerance [113]. In maize roots, Zn deficiency produced a massive loss of DNA methylation, mostly in TEs associated with up- and downregulated genes. Among these genes, several maintenance DNA methylation enzymes were downregulated, and a *ROS1* homolog was upregulated [114]. *Arabidopsis* wild-type seedlings grown under low phosphate (Pi) levels showed increases in DNA methylation levels concomitant with upregulation of transcript levels of all DNA methyltransferases (except for *CMT3*) and, to a lesser extent, *ROS1* and *DML2* transcripts, but exhibited downregulation of *DML3*, compared with high Pi conditions [115]. An *rdd* mutant showed a small increase in 5-meC, both in low and high Pi conditions, when compared to wild-type plants [115].

Active DNA demethylation is also involved in salt tolerance and responses to salt stress. The salt-tolerant rice variety Pokkali, but not the salt-sensitive variety IR29, showed decreased DNA methylation levels after salt stress, accompanied by induction in expression of *DNG701* and *DNG710* genes, encoding 5-meC DNA glycosylases, as well as demethylation of transposon Ty3-gypsy and a telomeric repetitive sequence [116]. Furthermore, recent evidence suggests that active DNA demethylation is involved in intergenerational transmission of a “stress memory” that facilitates rapid adaptation to short-term environmental fluctuations, a phenomenon known as ’priming’. Thus, hyperosmotic stress memory in *Arabidopsis* plants exposed to salt stress during their vegetative development is transiently transmitted to subsequent generations and is associated with changes in DNA methylation, primarily at non-CG sites located in intergenic TE-related sequences, and transcription levels of genes associated with environmental stress [117]. Salt stress tolerance and DNA methylation changes were preferentially transmitted through the female germline, but paternal memory transmission was restored in *dme* mutants, suggesting that active DNA demethylation in male gametes is essential to inhibit paternal inheritance of hyperosmotic priming responses [117]. 

Several studies have reported changes in DNA methylation levels and/or expression of DML homologs in different species subject to additional types of abiotic stresses such as drought [118,119,120], high or low temperatures [121,122], continuous cropping [123], and exposure to heavy metals [124] or ionizing radiation [125]. Active DNA demethylation has also been involved in the plant response to abscisic acid (ABA), a phytohormone that plays a crucial role in coordinating various signal transduction pathways during abiotic stresses. Thus, *Arabidopsis ros1* mutants are hypersensitive to ABA during early seedling development and ROS1 is required to demethylate and activate a subset of ABA-inducible genes [126]. Factors involved in regulation of active DNA demethylation during responses to abiotic stress are poorly understood, although microRNAs (miRNAs) might play a role. Thus, *Arabidopsis* plants overexpressing *miR402,* a regulatory miRNA induced under different stress conditions, exhibited downregulation of *DML3* expression and displayed earlier seed germination than wild-type plants under salt and cold stress, a phenotype that was also observed in *dml3* mutant plants [127].

## 5. Future Perspectives

Recent years have witnessed significant advances in our understanding of the molecular mechanisms and biological roles of active DNA demethylation in plants. However, there are still many open questions and important aspects that remain to be elucidated. For example, additional players in the demethylation pathway initiated by DML proteins, such as the DNA polymerase(s) involved in gap-filling, are yet to be identified. On the other hand, recent discoveries suggest that a complex web of regulatory mechanisms is required to balance methylation and demethylation processes, likely involving still unknown components. In this regard, the identification of the DNA repair factor DDB2 as a player in regulating DNA demethylation raises the question of how this process is coordinated with DNA repair pathways, particularly since potentially toxic DNA intermediates are generated during demethylation. Furthermore, the likely, but often overlooked, role of DML proteins in repairing T:G mispairs arising from 5-meC spontaneous deamination remains virtually unexplored. An additional area that needs to be further studied is the role that chromatin modifications and/or recruiting factors play in targeting 5-meC DNA glycosylases to specific genome regions. It is still unknown whether DML proteins are able to recognize specific histone modifications, either directly or through their interaction with recruiting factors. Furthermore, the possible role of small RNAs in targeting DNA demethylation is still an open question.

Advances in the study of basic molecular mechanisms of DNA demethylation will have to be complemented with additional studies to establish its specific roles in plant physiology. As efficient epigenetic erasers, it is likely that plant 5-meC DNA glycosylases play still unidentified functions in plant development and responses to environmental signals, including stress. In this area, studies in non-model plants will be increasingly important, particularly when addressing issues such as transgenerational transmission of stress memory or the generation and maintenance of epiallelic variation, which may have major impacts in crop productivity. 

Finally, our increasing knowledge about 5-meC DNA glycosylases may have also a significant impact in the emerging field of epigenetic editing, with potential applications not necessarily restricted to plants. Since no direct mechanism to excise 5-meC has apparently evolved in animals, it may be possible to use plant DML proteins to modify DNA methylation in human cells. Unlike TET-mediated oxidation, such direct excision would avoid generation of 5-meC derivatives that may have epigenetic roles on their own. Some recent results support the feasibility of using plant 5-meC DNA glycosylases as molecular tools to modify cell methylomes. Thus, overexpression of *Arabidopsis* DME in human cells causes genome-wide DNA methylation changes and significant alterations in the cellular phenotype, both in noncancerous [128] and cancer cells [129]. Furthermore, it has been reported that a fusion protein containing the catalytic domain of *Arabidopsis* ROS1 and the DNA-binding domain of yeast GAL4 can be targeted for specific demethylation and reactivation of a methylation-silenced reporter gene in human cells [130]. In summary, it is most likely that future research will generate new and exciting results on the molecular biology of plant active DNA demethylation and its physiological roles, as well as promising applications in emerging epigenetic technologies.

## References

[1] Law J.A., Jacobsen S.E. (2010). Establishing, maintaining and modifying DNA methylation patterns in plants and animals. Nat. Rev. Genet..

[2] Goll M.G., Bestor T.H. (2005). Eukaryotic cytosine methyltransferases. Annu. Rev. Biochem..

[3] Chan S.W., Henderson I.R., Jacobsen S.E. (2005). Gardening the genome: DNA methylation in Arabidopsis thaliana. Nat. Rev. Genet..

[4] Zemach A., Kim M.Y., Hsieh P.H., Coleman-Derr D., Eshed-Williams L., Thao K., Harmer S.L., Zilberman D. (2013). The Arabidopsis nucleosome remodeler DDM1 allows DNA methyltransferases to access H1-containing heterochromatin. Cell.

[5] Sasaki T., Kobayashi A., Saze H., Kakutani T. (2012). RNAi-independent de novo DNA methylation revealed in Arabidopsis mutants of chromatin remodeling gene DDM1. Plant J..

[6] Kress C., Thomassin H., Grange T. (2001). Local DNA demethylation in vertebrates: How could it be performed and targeted?. FEBS Lett..

[7] Ooi S.K., Bestor T.H. (2008). The colorful history of active DNA demethylation. Cell.

[8] Wu X., Zhang Y. (2017). TET-mediated active DNA demethylation: Mechanism, function and beyond. Nat. Rev. Genet..

[9] Roldan-Arjona T., Ariza R.R., Grosjean H. (2009). DNA demethylation. DNA and RNA modification Enzymes: Comparative Structure, Mechanism, Functions, Cellular Interactions and Evolution.

[10] Zhu J.K. (2009). Active DNA demethylation mediated by DNA glycosylases. Annu. Rev. Genet..

[11] Gong Z., Morales-Ruiz T., Ariza R.R., Roldan-Arjona T., David L., Zhu J.K. (2002). ROS1, a repressor of transcriptional gene silencing in Arabidopsis, encodes a DNA glycosylase/lyase. Cell.

[12] Morales-Ruiz T., Ortega-Galisteo A.P., Ponferrada-Marin M.I., Martinez-Macias M.I., Ariza R.R., Roldan-Arjona T. (2006). *DEMETER* and *REPRESSOR OF SILENCING 1* encode 5-methylcytosine DNA glycosylases. Proc. Natl. Acad. Sci. USA.

[13] Agius F., Kapoor A., Zhu J.K. (2006). Role of the Arabidopsis DNA glycosylase/lyase ROS1 in active DNA demethylation. Proc. Natl. Acad. Sci. USA.

[14] Choi Y., Gehring M., Johnson L., Hannon M., Harada J.J., Goldberg R.B., Jacobsen S.E., Fischer R.L. (2002). DEMETER, a DNA glycosylase domain protein, is required for endosperm gene imprinting and seed viability in Arabidopsis. Cell.

[15] Penterman J., Zilberman D., Huh J.H., Ballinger T., Henikoff S., Fischer R.L. (2007). DNA demethylation in the Arabidopsis genome. Proc. Natl. Acad. Sci. USA.

[16] Ortega-Galisteo A.P., Morales-Ruiz T., Ariza R.R., Roldan-Arjona T. (2008). Arabidopsis DEMETER-LIKE proteins DML2 and DML3 are required for appropriate distribution of DNA methylation marks. Plant Mol. Biol..

[17] Zhu J., Kapoor A., Sridhar V.V., Agius F., Zhu J.K. (2007). The DNA glycosylase/lyase ROS1 functions in pruning DNA methylation patterns in Arabidopsis. Curr. Biol..

[18] Gehring M., Huh J.H., Hsieh T.F., Penterman J., Choi Y., Harada J.J., Goldberg R.B., Fischer R.L. (2006). DEMETER DNA glycosylase establishes *MEDEA* polycomb gene self-imprinting by allele-specific demethylation. Cell.

[19] Calarco J.P., Borges F., Donoghue M.T., Van Ex F., Jullien P.E., Lopes T., Gardner R., Berger F., Feijo J.A., Becker J.D. (2012). Reprogramming of DNA methylation in pollen guides epigenetic inheritance via small RNA. Cell.

[20] Ibarra C.A., Feng X., Schoft V.K., Hsieh T.F., Uzawa R., Rodrigues J.A., Zemach A., Chumak N., Machlicova A., Nishimura T. (2012). Active DNA demethylation in plant companion cells reinforces transposon methylation in gametes. Science.

[21] Kawakatsu T., Nery J.R., Castanon R., Ecker J.R. (2017). Dynamic DNA methylation reconfiguration during seed development and germination. Genome Biol..

[22] Lang Z., Wang Y., Tang K., Tang D., Datsenka T., Cheng J., Zhang Y., Handa A.K., Zhu J.K. (2017). Critical roles of DNA demethylation in the activation of ripening-induced genes and inhibition of ripening-repressed genes in tomato fruit. Proc. Natl. Acad. Sci. USA.

[23] Liu R., How-Kit A., Stammitti L., Teyssier E., Rolin D., Mortain-Bertrand A., Halle S., Liu M., Kong J., Wu C. (2015). A DEMETER-like DNA demethylase governs tomato fruit ripening. Proc. Natl. Acad. Sci. USA.

[24] Pandey G., Sharma N., Sahu P.P., Prasad M. (2016). Chromatin-Based Epigenetic Regulation of Plant Abiotic Stress Response. Curr. Genomics.

[25] Yu A., Lepere G., Jay F., Wang J., Bapaume L., Wang Y., Abraham A.L., Penterman J., Fischer R.L., Voinnet O. (2013). Dynamics and biological relevance of DNA demethylation in Arabidopsis antibacterial defense. Proc. Natl. Acad. Sci. USA.

[26] Nash H.M., Bruner S.D., Scharer O.D., Kawate T., Addona T.A., Spooner E., Lane W.S., Verdine G.L. (1996). Cloning of a yeast 8-oxoguanine DNA glycosylase reveals the existence of a base-excision DNA-repair protein superfamily. Curr. Biol..

[27] Ponferrada-Marín M.I., Parrilla-Doblas J.T., Roldán-Arjona T., Ariza R.R. (2011). A discontinuous DNA glycosylase domain in a family of enzymes that excise 5-methylcytosine. Nucleic. Acids. Res..

[28] Mok Y.G., Uzawa R., Lee J., Weiner G.M., Eichman B.F., Fischer R.L., Huh J.H. (2010). Domain structure of the DEMETER 5-methylcytosine DNA glycosylase. Proc. Natl. Acad. Sci. USA.

[29] Krokan H.E., Standal R., Slupphaug G. (1997). DNA glycosylases in the base excision repair of DNA. Biochem. J..

[30] Parrilla-Doblas J.T., Ponferrada-Marin M.I., Roldan-Arjona T., Ariza R.R. (2013). Early steps of active DNA demethylation initiated by ROS1 glycosylase require three putative helix-invading residues. Nucleic Acids Res..

[31] Buzas D.M. (2016). Emerging links between iron-sulfur clusters and 5-methylcytosine base excision repair in plants. Genes Genet. Syst..

[32] Ponferrada-Marín M.I., Martínez-Macías M.I., Morales-Ruiz T., Roldán-Arjona T., Ariza R.R. (2010). Methylation-independent DNA binding modulates specificity of repressor of silencing 1 (ROS1) and facilitates demethylation in long substrates. J. Biol. Chem..

[33] Ponferrada-Marín M.I., Roldán-Arjona T., Ariza R.R. (2012). Demethylation initiated by ROS1 glycosylase involves random sliding along DNA. Nucleic Acids Res..

[34] Hong S., Hashimoto H., Kow Y.W., Zhang X., Cheng X. (2014). The carboxy-terminal domain of ROS1 is essential for 5-methylcytosine DNA glycosylase activity. J. Mol. Biol..

[35] Ponferrada-Marín M.I., Roldán-Arjona T., Ariza R.R. (2009). ROS1 5-methylcytosine DNA glycosylase is a slow-turnover catalyst that initiates DNA demethylation in a distributive fashion. Nucleic Acids Res..

[36] Dodson M.L., Michaels M.L., Lloyd R.S. (1994). Unified catalytic mechanism for DNA glycosylases. J. Biol. Chem..

[37] Martínez-Macías M.I., Qian W., Miki D., Pontes O., Liu Y., Tang K., Liu R., Morales-Ruiz T., Ariza R.R., Roldán-Arjona T. (2012). A DNA 3′ phosphatase functions in active DNA demethylation in Arabidopsis. Mol. Cell.

[38] Córdoba-Cañero D., Roldan-Arjona T., Ariza R.R. (2014). Arabidopsis ZDP DNA 3′-phosphatase and ARP endonuclease function in 8-oxoG repair initiated by FPG and OGG1 DNA glycosylases. Plant J..

[39] Barbado C., Córdoba-Cañero D., Ariza R.R., Roldan-Arjona T. (2018). Nonenzymatic release of N7-methylguanine channels repair of abasic sites into an AP endonuclease-independent pathway in Arabidopsis. Proc. Natl. Acad. Sci. USA.

[40] Li J., Liang W., Li Y., Qian W. (2018). Apurinic/apyrimidinic endonuclease2 and zinc finger dna 3′-phosphoesterase play overlapping roles in the maintenance of epigenome and genome stability. Plant Cell.

[41] Suh D., Wilson D.M., Povirk L.F. (1997). 3′-phosphodiesterase activity of human apurinic/apyrimidinic endonuclease at DNA double-strand break ends. Nucleic Acids Res..

[42] Li Y., Córdoba-Cañero D., Qian W., Zhu X., Tang K., Zhang H., Ariza R.R., Roldán-Arjona T., Zhu J.K. (2015). An AP endonuclease functions in active DNA demethylation and gene imprinting in *Arabidopsis*. PLoS Genet.

[43] Lee J., Jang H., Shin H., Choi W.L., Mok Y.G., Huh J.H. (2015). AP endonucleases process 5-methylcytosine excision intermediates during active DNA demethylation in Arabidopsis. Nucleic Acids Res..

[44] Fortini P., Dogliotti E. (2007). Base damage and single-strand break repair: Mechanisms and functional significance of short- and long-patch repair subpathways. DNA Repair (Amst).

[45] Córdoba-Cañero D., Morales-Ruiz T., Roldán-Arjona T., Ariza R.R. (2009). Single-nucleotide and long-patch base excision repair of DNA damage in plants. Plant J..

[46] Kubota Y., Nash R.A., Klungland A., Schar P., Barnes D.E., Lindahl T. (1996). Reconstitution of DNA base excision-repair with purified human proteins: Interaction between DNA polymerase b and the XRCC1 protein. EMBO J..

[47] Podlutsky A.J., Dianova II., Podust V.N., Bohr V.A., Dianov G.L. (2001). Human DNA polymerase beta initiates DNA synthesis during long-patch repair of reduced AP sites in DNA. Embo J..

[48] Uchiyama Y., Kimura S., Yamamoto T., Ishibashi T., Sakaguchi K. (2004). Plant DNA polymerase l, a DNA repair enzyme that functions in plant meristematic and meiotic tissues. Eur. J. Biochem..

[49] Amoroso A., Concia L., Maggio C., Raynaud C., Bergounioux C., Crespan E., Cella R., Maga G. (2011). Oxidative DNA damage bypass in Arabidopsis thaliana requires DNA Polymerase l and Proliferating Cell Nuclear Antigen 2. Plant Cell.

[50] Roy S., Choudhury S.R., Singh S.K., Das K.P. (2011). AtPoll, a homolog of mammalian DNA polymerase l in *Arabidopsis thaliana*, is involved in the repair of UV-B induced DNA damage through the dark repair pathway. Plant Cell Physiol..

[51] Balakrishnan L., Bambara R.A. (2013). Flap endonuclease 1. Annu. Rev. Biochem..

[52] Zhang J., Xie S., Zhu J.K., Gong Z. (2016). Requirement for flap endonuclease 1 (FEN1) to maintain genomic stability and transcriptional gene silencing in Arabidopsis. Plant J..

[53] Cappelli E., Taylor R., Cevasco M., Abbondandolo A., Caldecott K., Frosina G. (1997). Involvement of XRCC1 and DNA ligase III gene products in DNA base excision repair. J. Biol. Chem..

[54] Sleeth K.M., Robson R.L., Dianov G.L. (2004). Exchangeability of mammalian DNA ligases between base excision repair pathways. Biochemistry.

[55] Bonatto D., Brendel M., Henriques J.A.P. (2005). A new group of plant-specific ATP-dependent DNA ligases identified by protein phylogeny, hydrophobic cluster analysis and 3-dimensional modelling. Funct. Plant Biol..

[56] Waterworth W.M., Kozak J., Provost C.M., Bray C.M., Angelis K.J., West C.E. (2009). DNA ligase 1 deficient plants display severe growth defects and delayed repair of both DNA single and double strand breaks. BMC Plant Biol..

[57] Waterworth W.M., Masnavi G., Bhardwaj R.M., Jiang Q., Bray C.M., West C.E. (2010). A higher plant DNA ligase is an important determinant of seed longevity. Plant J..

[58] West C.E., Waterworth W.M., Jiang Q., Bray C.M. (2000). Arabidopsis DNA ligase IV is induced by gamma-irradiation and interacts with an Arabidopsis homologue of the double strand break repair protein XRCC4. Plant J..

[59] Córdoba-Cañero D., Roldán-Arjona T., Ariza R.R. (2011). Arabidopsis ARP endonuclease functions in a branched base excision DNA repair pathway completed by LIG1. Plant J..

[60] Li Y., Duan C.G., Zhu X., Qian W., Zhu J.K. (2015). A DNA ligase required for active DNA demethylation and genomic imprinting in Arabidopsis. Cell Res..

[61] Andreuzza S., Li J., Guitton A.E., Faure J.E., Casanova S., Park J.S., Choi Y., Chen Z., Berger F. (2009). DNA LIGASE I exerts a maternal effect on seed development in Arabidopsis thaliana. Development.

[62] Martinez-Macías M.I., Cordoba-Cañero D., Ariza R.R., Roldan-Arjona T. (2013). The DNA repair protein XRCC1 functions in the plant DNA demethylation pathway by stimulating cytosine methylation (5-meC) excision, gap tailoring, and DNA ligation. J. Biol. Chem..

[63] Tang K., Lang Z., Zhang H., Zhu J.K. (2016). The DNA demethylase ROS1 targets genomic regions with distinct chromatin modifications. Nat. Plants.

[64] Qian W., Miki D., Zhang H., Liu Y., Zhang X., Tang K., Kan Y., La H., Li X., Li S. (2012). A histone acetyltransferase regulates active DNA demethylation in Arabidopsis. Science.

[65] Qian W., Miki D., Lei M., Zhu X., Zhang H., Liu Y., Li Y., Lang Z., Wang J., Tang K. (2014). Regulation of active DNA demethylation by an alpha-crystallin domain protein in Arabidopsis. Mol. Cell.

[66] Lang Z., Lei M., Wang X., Tang K., Miki D., Zhang H., Mangrauthia S.K., Liu W., Nie W., Ma G. (2015). The methyl-CpG-binding protein MBD7 facilitates active DNA demethylation to limit DNA hyper-methylation and transcriptional gene silencing. Mol. Cell.

[67] Duan C.G., Wang X., Xie S., Pan L., Miki D., Tang K., Hsu C.C., Lei M., Zhong Y., Hou Y.J. (2017). A pair of transposon-derived proteins function in a histone acetyltransferase complex for active DNA demethylation. Cell Res..

[68] Zheng X., Pontes O., Zhu J., Miki D., Zhang F., Li W.X., Iida K., Kapoor A., Pikaard C.S., Zhu J.K. (2008). ROS3 is an RNA-binding protein required for DNA demethylation in Arabidopsis. Nature.

[69] Lister R., O’Malley R.C., Tonti-Filippini J., Gregory B.D., Berry C.C., Millar A.H., Ecker J.R. (2008). Highly integrated single-base resolution maps of the epigenome in Arabidopsis. Cell.

[70] Huettel B., Kanno T., Daxinger L., Aufsatz W., Matzke A.J., Matzke M. (2006). Endogenous targets of RNA-directed DNA methylation and Pol IV in Arabidopsis. EMBO J..

[71] Mathieu O., Reinders J., Caikovski M., Smathajitt C., Paszkowski J. (2007). Transgenerational stability of the Arabidopsis epigenome is coordinated by CG methylation. Cell.

[72] Penterman J., Uzawa R., Fischer R.L. (2007). Genetic interactions between DNA demethylation and methylation in Arabidopsis. Plant Physiol..

[73] Gao Z., Liu H.L., Daxinger L., Pontes O., He X., Qian W., Lin H., Xie M., Lorkovic Z.J., Zhang S. (2010). An RNA polymerase II- and AGO4-associated protein acts in RNA-directed DNA methylation. Nature.

[74] Li X., Qian W., Zhao Y., Wang C., Shen J., Zhu J.K., Gong Z. (2012). Antisilencing role of the RNA-directed DNA methylation pathway and a histone acetyltransferase in Arabidopsis. Proc. Natl. Acad Sci. USA.

[75] Lei M., Zhang H., Julian R., Tang K., Xie S., Zhu J.K. (2015). Regulatory link between DNA methylation and active demethylation in Arabidopsis. Proc. Natl. Acad Sci. USA.

[76] Williams B.P., Pignatta D., Henikoff S., Gehring M. (2015). Methylation-sensitive expression of a DNA demethylase gene serves as an epigenetic rheostat. PLoS Genet..

[77] Wittschieben B.O., Iwai S., Wood R.D. (2005). DDB1-DDB2 (xeroderma pigmentosum group E) protein complex recognizes a cyclobutane pyrimidine dimer, mismatches, apurinic/apyrimidinic sites, and compound lesions in DNA. J. Biol. Chem..

[78] Schalk C., Drevensek S., Kramdi A., Kassam M., Ahmed I., Cognat V., Graindorge S., Bergdoll M., Baumberger N., Heintz D. (2016). DNA DAMAGE BINDING PROTEIN2 Shapes the DNA Methylation Landscape. Plant Cell.

[79] Córdoba-Cañero D., Cognat V., Ariza R.R., Roldan Arjona T., Molinier J. (2017). Dual control of ROS1-mediated active DNA demethylation by DNA damage-binding protein 2 (DDB2). Plant J..

[80] Luo D., Bernard D.G., Balk J., Hai H., Cui X. (2012). The DUF59 family gene AE7 acts in the cytosolic iron-sulfur cluster assembly pathway to maintain nuclear genome integrity in Arabidopsis. Plant Cell.

[81] Nakamura M., Buzas D.M., Kato A., Fujita M., Kurata N., Kinoshita T. (2013). The role of Arabidopsis thaliana NAR1, a cytosolic iron-sulfur cluster assembly component, in gametophytic gene expression and oxidative stress responses in vegetative tissue. New Phytol..

[82] Buzas D.M., Nakamura M., Kinoshita T. (2014). Epigenetic role for the conserved Fe-S cluster biogenesis protein AtDRE2 in Arabidopsis thaliana. Proc. Natl. Acad Sci. USA.

[83] Duan C.G., Wang X., Tang K., Zhang H., Mangrauthia S.K., Lei M., Hsu C.C., Hou Y.J., Wang C., Li Y. (2015). MET18 Connects the cytosolic iron-sulfur cluster assembly pathway to active dna demethylation in arabidopsis. PLoS Genet..

[84] Han Y.F., Huang H.W., Li L., Cai T., Chen S., He X.J. (2015). The cytosolic iron-sulfur cluster assembly protein mms19 regulates transcriptional gene silencing, dna repair, and flowering time in arabidopsis. PLoS ONE.

[85] Tran R.K., Zilberman D., de Bustos C., Ditt R.F., Henikoff J.G., Lindroth A.M., Delrow J., Boyle T., Kwong S., Bryson T.D. (2005). Chromatin and siRNA pathways cooperate to maintain DNA methylation of small transposable elements in Arabidopsis. Genome Biol..

[86] Stroud H., Do T., Du J., Zhong X., Feng S., Johnson L., Patel D.J., Jacobsen S.E. (2014). Non-CG methylation patterns shape the epigenetic landscape in Arabidopsis. Nat. Struct. Mol. Biol..

[87] La H., Ding B., Mishra G.P., Zhou B., Yang H., Bellizzi Mdel R., Chen S., Meyers B.C., Peng Z., Zhu J.K. (2011). A 5-methylcytosine DNA glycosylase/lyase demethylates the retrotransposon Tos17 and promotes its transposition in rice. Proc. Natl. Acad. Sci. USA.

[88] Yamamuro C., Miki D., Zheng Z., Ma J., Wang J., Yang Z., Dong J., Zhu J.K. (2014). overproduction of stomatal lineage cells in arabidopsis mutants defective in active dna demethylation. Nat. Commun..

[89] Liu Q., Wang J., Miki D., Xia R., Yu W., He J., Zheng Z., Zhu J.K., Gong Z. (2010). DNA replication factor c1 mediates genomic stability and transcriptional gene silencing in Arabidopsis. Plant Cell.

[90] Huh J.H., Bauer M.J., Hsieh T.F., Fischer R.L. (2008). Cellular programming of plant gene imprinting. Cell.

[91] Park K., Kim M.Y., Vickers M., Park J.S., Hyun Y., Okamoto T., Zilberman D., Fischer R.L., Feng X., Choi Y. (2016). DNA demethylation is initiated in the central cells of Arabidopsis and rice. Proc. Natl. Acad. Sci. USA.

[92] Kinoshita T., Miura A., Choi Y., Kinoshita Y., Cao X., Jacobsen S.E., Fischer R.L., Kakutani T. (2004). one-way control of FWA imprinting in arabidopsis endosperm by DNA methylation. Science.

[93] Jullien P.E., Kinoshita T., Ohad N., Berger F. (2006). Maintenance of DNA methylation during the Arabidopsis life cycle is essential for parental imprinting. Plant Cell.

[94] Gehring M., Bubb K.L., Henikoff S. (2009). Extensive demethylation of repetitive elements during seed development underlies gene imprinting. Science.

[95] Hsieh T.F., Shin J., Uzawa R., Silva P., Cohen S., Bauer M.J., Hashimoto M., Kirkbride R.C., Harada J.J., Zilberman D. (2011). Regulation of imprinted gene expression in Arabidopsis endosperm. Proc. Natl. Acad. Sci. USA.

[96] Hsieh T.F., Ibarra C.A., Silva P., Zemach A., Eshed-Williams L., Fischer R.L., Zilberman D. (2009). Genome-wide demethylation of Arabidopsis endosperm. Science.

[97] Schoft V.K., Chumak N., Choi Y., Hannon M., Garcia-Aguilar M., Machlicova A., Slusarz L., Mosiolek M., Park J.S., Park G.T. (2011). Function of the DEMETER DNA glycosylase in the Arabidopsis thaliana male gametophyte. Proc. Natl. Acad. Sci. USA.

[98] Park J.S., Frost J.M., Park K., Ohr H., Park G.T., Kim S., Eom H., Lee I., Brooks J.S., Fischer R.L. (2017). Control of DEMETER DNA demethylase gene transcription in male and female gamete companion cells in Arabidopsis thaliana. Pro. Natl. Acad. Sci. USA.

[99] Slotkin R.K., Vaughn M., Borges F., Tanurdzic M., Becker J.D., Feijo J.A., Martienssen R.A. (2009). Epigenetic reprogramming and small RNA silencing of transposable elements in pollen. Cell.

[100] Martinez G., Panda K., Kohler C., Slotkin R.K. (2016). Silencing in sperm cells is directed by RNA movement from the surrounding nurse cell. Nat. Plants.

[101] Zemach A., Kim M.Y., Silva P., Rodrigues J.A., Dotson B., Brooks M.D., Zilberman D. (2010). Local DNA hypomethylation activates genes in rice endosperm. Proc. Natl. Acad. Sci. USA.

[102] Ono A., Yamaguchi K., Fukada-Tanaka S., Terada R., Mitsui T., Iida S. (2012). A null mutation of ROS1a for DNA demethylation in rice is not transmittable to progeny. Plant J..

[103] Kim M.Y., Ono A., Scholten S., Kinoshita T., Zilberman D., Okamoto T., Fischer R.L. (2019). DNA demethylation by ROS1a in rice vegetative cells promotes methylation in sperm. Proc. Natl. Acad. Sci. USA.

[104] Wang P., Xia H., Zhang Y., Zhao S., Zhao C., Hou L., Li C., Li A., Ma C., Wang X. (2015). Genome-wide high-resolution mapping of DNA methylation identifies epigenetic variation across embryo and endosperm in Maize (Zea may). BMC Genomics.

[105] Wen S., Wen N., Pang J., Langen G., Brew-Appiah R.A., Mejias J.H., Osorio C., Yang M., Gemini R., Moehs C.P. (2012). Structural genes of wheat and barley 5-methylcytosine DNA glycosylases and their potential applications for human health. Proc. Natl. Acad. Sci. USA.

[106] Cheng J., Niu Q., Zhang B., Chen K., Yang R., Zhu J.K., Zhang Y., Lang Z. (2018). Downregulation of RdDM during strawberry fruit ripening. Genome Biol..

[107] Huang H., Liu R., Niu Q., Tang K., Zhang B., Zhang H., Chen K., Zhu J.K., Lang Z. (2019). Global increase in DNA methylation during orange fruit development and ripening. Proc. Natl. Acad. Sci. USA.

[108] Satge C., Moreau S., Sallet E., Lefort G., Auriac M.C., Rembliere C., Cottret L., Gallardo K., Noirot C., Jardinaud M.F. (2016). Reprogramming of DNA methylation is critical for nodule development in Medicago truncatula. Nat. Plants.

[109] Rodriguez-Negrete E., Lozano-Duran R., Piedra-Aguilera A., Cruzado L., Bejarano E.R., Castillo A.G. (2013). Geminivirus Rep protein interferes with the plant DNA methylation machinery and suppresses transcriptional gene silencing. New Phytol..

[110] Le T.N., Schumann U., Smith N.A., Tiwari S., Au P., Zhu Q.H., Taylor J., Kazan K., Llewellyn D.J., Zhang R. (2014). DNA demethylases target promoter transposable elements to positively regulate stress responsive genes in Arabidopsis. Genome Biol..

[111] Schumann U., Lee J., Kazan K., Ayliffe M., Wang M.B. (2017). DNA-Demethylase Regulated Genes Show Methylation-Independent Spatiotemporal Expression Patterns. Front Plant Sci..

[112] Lopez Sanchez A., Stassen J.H., Furci L., Smith L.M., Ton J. (2016). The role of DNA (de)methylation in immune responsiveness of Arabidopsis. Plant J..

[113] Chen X., Schonberger B., Menz J., Ludewig U. (2018). Plasticity of DNA methylation and gene expression under zinc deficiency in Arabidopsis roots. Plant Cell Physiol..

[114] Mager S., Ludewig U. (2018). Massive Loss of DNA Methylation in Nitrogen-, but Not in Phosphorus-Deficient Zea mays Roots Is Poorly Correlated With Gene Expression Differences. Front Plant Sci..

[115] Yong-Villalobos L., Gonzalez-Morales S.I., Wrobel K., Gutierrez-Alanis D., Cervantes-Perez S.A., Hayano-Kanashiro C., Oropeza-Aburto A., Cruz-Ramirez A., Martinez O., Herrera-Estrella L. (2015). Methylome analysis reveals an important role for epigenetic changes in the regulation of the Arabidopsis response to phosphate starvation. Proc. Natl. Acad. Sci. USA.

[116] Ferreira L.J., Azevedo V., Maroco J., Oliveira M.M., Santos A.P. (2015). Salt tolerant and sensitive rice varieties display differential methylome flexibility under salt stress. PLoS ONE.

[117] Wibowo A., Becker C., Marconi G., Durr J., Price J., Hagmann J., Papareddy R., Putra H., Kageyama J., Becker J. (2016). Hyperosmotic stress memory in Arabidopsis is mediated by distinct epigenetically labile sites in the genome and is restricted in the male germline by DNA glycosylase activity. Elife.

[118] Kapazoglou A., Drosou V., Argiriou A., Tsaftaris A.S. (2013). The study of a barley epigenetic regulator, HvDME, in seed development and under drought. BMC Plant Biol..

[119] Chwialkowska K., Nowakowska U., Mroziewicz A., Szarejko I., Kwasniewski M. (2016). Water-deficiency conditions differently modulate the methylome of roots and leaves in barley (Hordeum vulgare L.). J. Exp. Bot..

[120] Xu P., Chen H., Jin J., Cai W. (2018). Single-base resolution methylome analysis shows epigenetic changes in Arabidopsis seedlings exposed to microgravity spaceflight conditions on board the SJ-10 recoverable satellite. NPJ Microgravity.

[121] Naydenov M., Baev V., Apostolova E., Gospodinova N., Sablok G., Gozmanova M., Yahubyan G. (2015). High-temperature effect on genes engaged in DNA methylation and affected by DNA methylation in Arabidopsis. Plant Physiol. Biochem..

[122] Zhang B., Tieman D.M., Jiao C., Xu Y., Chen K., Fei Z., Giovannoni J.J., Klee H.J. (2016). Chilling-induced tomato flavor loss is associated with altered volatile synthesis and transient changes in DNA methylation. Proc. Natl. Acad. Sci. USA.

[123] Liang X., Hou X., Li J., Han Y., Zhang Y., Feng N., Du J., Zhang W., Zheng D., Fang S. (2019). High-resolution DNA methylome reveals that demethylation enhances adaptability to continuous cropping comprehensive stress in soybean. BMC Plant Biol..

[124] Ou X., Zhang Y., Xu C., Lin X., Zang Q., Zhuang T., Jiang L., von Wettstein D., Liu B. (2012). Transgenerational inheritance of modified DNA methylation patterns and enhanced tolerance induced by heavy metal stress in rice (Oryza sativa L.). PLoS ONE.

[125] Xu W., Wang T., Xu S., Xu S., Wu L., Wu Y., Bian P. (2015). Radiation-induced epigenetic bystander effects demonstrated in Arabidopsis thaliana. Radiat. Res..

[126] Kim J.S., Lim J.Y., Shin H., Kim B.G., Yoo S.D., Kim W.T., Huh J.H. (2019). ROS1-dependent DNA demethylation is required for ABA-inducible NIC3 expression. Plant Physiol..

[127] Kim J.Y., Kwak K.J., Jung H.J., Lee H.J., Kang H. (2010). MicroRNA402 affects seed germination of Arabidopsis thaliana under stress conditions via targeting DEMETER-LIKE Protein3 mRNA. Plant Cell Physiol..

[128] Mok Y.G., Choi K.Y., Hong S.H., Huh J.H. (2017). DEMETER plant DNA demethylase induces antiviral response by interferon signalling in animal cells. Sci. Rep..

[129] Morales-Ruiz T., Garcia-Ortiz M.V., Devesa-Guerra I., Raya-Ruiz L., Tejedor J.R., Bayon G.F., Sierra M.I., Fraga M.F., Ariza R.R., Roldan-Arjona T. (2018). DNA methylation reprogramming of human cancer cells by expression of a plant 5-methylcytosine DNA glycosylase. Epigenetics.

[130] Parrilla-Doblas J.T., Ariza R.R., Roldan-Arjona T. (2017). Targeted DNA demethylation in human cells by fusion of a plant 5-methylcytosine DNA glycosylase to a sequence-specific DNA binding domain. Epigenetics.

